# Prevalence of HIV, syphilis, and assessment of the social and structural determinants of sexual risk behaviour and health service utilisation among MSM and transgender women in Terai highway districts of Nepal: findings based on an integrated biological and behavioural surveillance survey using respondent driven sampling

**DOI:** 10.1186/s12879-020-05122-3

**Published:** 2020-06-08

**Authors:** Margrethe Storm, Keshab Deuba, Jose Damas, Upendra Shrestha, Bir Rawal, Rajan Bhattarai, Gaetano Marrone

**Affiliations:** 1 Department of Global Public Health, Karolinska Institutet, Stockholm, Sweden; 2 National Centre for AIDS and STD Control, Kathmandu, Nepal; 3 Save the Children International, Kathmandu, Nepal

**Keywords:** MSM, Transgender women, HIV, STI, Nepal, Sexual risk behaviour, Health care service uptake, Structural factors

## Abstract

**Background:**

Men who have sex with men (MSM) and transgender people are disproportionately affected by HIV and sexually transmitted infections. MSM and transgender people in Nepal experience considerable discrimination and marginalisation, they are subject to abuse from legal authorities and suffer from mental health issues. These social and structural factors can lead to increased sexual risk behaviour, barriers to accessing health care and result in adverse health outcomes. This study aims to assess the prevalence of HIV and syphilis, and how individual and socio-structural factors influence sexual risk behaviour and health care service uptake, among MSM and transgender women in the Terai highway districts of Nepal.

**Methods:**

A cross-sectional survey was conducted in June 2016 in eight Terai highway districts of Nepal, recruiting 340 MSM and transgender women through respondent driven sampling. The primary outcome variables were HIV and syphilis prevalence. The secondary outcome variables were sexual risk behaviour and health care service uptake. Logistic regression models were used to assess the individual and socio-structural determinants of sexual risk behaviour and health care service uptake.

**Results:**

The prevalence of HIV among MSM was 5%, whereas it was 13% in transgender women. The prevalence of active syphilis was 4% in MSM and 11% among transgender women. Among transgender women, 76% were involved in sex work, and 51% had experienced discrimination in one or more settings. In multivariable analysis, having visited an outreach centre was positively associated with condom use in the last sexual encounter among both MSM (AOR: 5.37, 95% CI: 2.42–11.94, *p* < 0.001) and transgender women (AOR: 2.37, 95% CI: 1.12–5.02, *p* = 0.025). Moreover, transgender women who reported being open towards family about sexual identity/behaviour were 2.4 more likely to have visited an outreach centre (AOR: 2.40, 95% CI: 1.04–5.57, *p* = 0.041).

**Conclusions:**

The high prevalence of HIV and syphilis, as well as indicators of marginalisation and discrimination among transgender women, highlights the increased burden transgender women in Nepal are facing and the need for tailored interventions. Moreover, since health care service uptake is an important factor in determining sexual risk behaviour among MSM and transgender women in Nepal, outreach services should be scaled up.

## Background

HIV/AIDS remains an important issue in the world with 1.7 million estimated new infections in 2018 [1]. Key populations such as men who have sex with men (MSM) and transgender people are disproportionately affected by the HIV epidemic and have a higher risk of infection either due to a higher level of risk behaviour, or their vulnerable position in society [2]. MSM is an epidemiological term to describe men who engage in sex with other men regardless of their sexual orientation or gender identity, whereas the term transgender is used to describe individuals whose gender identity and/or expression does not correspond with the sex assigned to them at birth [2]. Globally, MSM are estimated to be 19 times more likely to be infected with HIV than the general population and HIV prevalence rates in transgender women are as high as 40% in some settings [3, 4]. In the South East Asia region, five countries (India, Indonesia, Myanmar, Nepal, and Thailand) account for 99% of the regional HIV burden [5].

Nepal is a low-income country with a population of 29 million people and consists of a mountainous region in the north and open terrain (Terai in local language) in the south [6]. Nepal has in the last 15 years experienced a decline in HIV prevalence, however, the HIV epidemic has evolved from being a “low prevalence” to a “concentrated epidemic”, i.e. HIV transmission largely occurs in one or more defined subpopulation but is not well established in the general population [7, 8]. In 2018, Nepal had an estimated adult HIV prevalence of 0.1% [9]. The key affected populations in Nepal include MSM, transgender people, people who inject drugs (PWID), male sex workers (MSW), female sex workers (FSW), and male labour migrants and their spouses [7]. Similarly to HIV, sexually transmitted infections (STIs) are also disproportionately represented in the key populations in Nepal [7, 10]. Nepal has in the last decade observed a decline in HIV prevalence among young MSM/transgender women in the Kathmandu valley which coincided with an increase in condom use in this group [11]. In 2017, the prevalence of HIV was 4.8 and 8.6% among MSM and transgender women, respectively, in the Kathmandu Valley [7].

The studies to date in Nepal have only focused on MSM and transgender women in the Kathmandu Valley, however key populations are scattered around the country, and there is limited data on MSM and transgender women in hard to reach areas of Nepal. The Terai highway districts of Nepal have a high population density and share an open border with India in the south, leading to an increased risk of transmission of HIV and STIs due to sex work and drug trafficking across the border [12]. Furthermore, there is limited data on transgender people in Nepal both with regards to health indicators, as well as estimates of population numbers, and transgender people are often subsumed under the MSM term. Although MSM and transgender women share a number of challenges, several studies indicate that transgender women face additional individual and socio-structural obstacles and experience a higher burden of adverse health conditions than MSM [4, 13].

Sexual risk behaviour is now recognized as multifaceted and composed of not only individual, but also social and structural factors such as social networks, stigma, discrimination, policies and laws [14, 15]. MSM and transgender women in Nepal face several social and structural challenges that significantly influence their health and standing in society, such as discrimination, lack of adequate legal protection, reduced social capital, and mental health issues [16–18]. These conditions can, either alone or together, lead to barriers to access to health care, increased sexual risk behaviour, and ultimately poor health outcomes [17, 18].

Nepal has made some progress in terms of the rights of lesbian, gay, bisexual and transgender (LGBT) with the Supreme Court ruling of 2007, which promoted the abolishment of discriminatory laws against sexual and gender minorities, same-sex marriage, and the official recognition of the third gender [10]. Furthermore, the new constitution of 2015 ensures the rights of sexual and gender minorities [16]. A number of non-governmental organisations (NGOs) also advocate the rights and health needs of the Nepalese LGBT community with the most prominent being the Blue Diamond Society (BDS) [10]. However, the implementation of the third gender, same-sex marriages, and prevention of discrimination in private and public settings are still subject to controversy [17, 19]. A study which assessed risk of HIV infection among MSM in Kathmandu found that a large proportion of the survey participants had experienced verbal, physical and sexual abuse which was associated with increased sexual risk behaviour in the bivariate analysis [20]. Furthermore, a recent study investigating the psychosocial well-being of MSM/transgender women found a high prevalence of mental health issues which was associated with increased sexual risk behaviour [19]. Sexual risk behaviour was connected to low social support, experienced violence and not participating in HIV prevention [19]. Another study that explored the underpinnings of psychosocial issues among MSM/transgender women in Nepal found that suicidal ideation was associated with perceived discrimination and increased with the extent of perceived discrimination [21].

Despite extensive research into the individual risk determinants of HIV/STIs among MSM and transgender women in Nepal, there is a lack of quantitative studies describing how socio-structural factors influence sexual risk behaviour and health service uptake among MSM and transgender women in Nepal. The objective of this study was to assess the HIV and STIs prevalence, sexual risk behaviour, health service uptake, and how it relates to individual and socio-structural factors among MSM and transgender women in the Terai highway districts in order to obtain information for planning of policies and programs for treatment and prevention of HIV and STIs in Nepal.

## Methods

### Study design and setting

This study was based on secondary analysis of data from a cross-sectional Integrated Biological and Behavioural Surveillance (IBBS) survey conducted in the Terai highway districts of Nepal in 2016 using respondent driven sampling (RDS). This study adheres to the STROBE-RDS guidelines for reporting of respondent-driven sampling studies [22]. The IBBS survey covered eight districts of the Terai region; Jhapa, Morang, Sunsari, Nawalparasi, Rupandehi, Kapilbastu, Kailali, and Kanchanpur. The districts were chosen based on previous mapping and size estimations reports of MSM and transgender women, as well as stakeholders’ suggestions. Survey facilities and clinics were set up in Itahari of Sunsari district, Bhairahawa of Rupandehi district, and Dhangadhi of Kailali district. The National Centre for AIDS and STD Control (NCASC) conducted the survey with technical and financial support from Save the Children International/ Global Fund Programs, and in collaboration with BDS and other community based organisations working in the area. A formative assessment was carried out prior to the survey to ensure that the sampling strategy, survey protocol, as well as sample size estimates were appropriate. Moreover, the formative assessment found that the RDS method could possibly lead to cross recruitment of survey participants in the adjoining districts, however given that the populations are geographically scattered it was more likely that respondents will refer others in the same region. Personal network size was assessed by the question: “How many MSM/transgender women do you know who also know you, who have been involved in sexual activities with men in the past 3 months? How many of these MSM/transgender women have you seen in the past month?”. Additional details of the survey design have been published in the IBBS report [23].

### Study population

Individuals were eligible to participate if they were 1) biological males (including participants who either identified themselves in a different gender than that assigned to them at birth or identified themselves belonging to transgender community), 2) aged 16 years or above, 3) reported they had sexual relations (either oral or anal) with another biological male at least once in the past year prior to the survey, 4) resided in one of the eight selected districts of the Terai highway, 5) had a valid referral coupon and 6) had not previously participated in the survey. The definition of a sex worker in the survey comprised biological males who had anal or oral sex with another male in exchange for money or other items in the past year prior to the survey.

### Sampling strategy

Participants were recruited using respondent driven sampling as both MSM and transgender women are hard to reach populations [24]. Starting with the purposive sampling of eight seeds from each of the selected survey districts, an additional seed was later added at the Bhairahawa facility to increase coverage. The seeds were selected to represent a diverse sample of sexual identities, age, caste, education, and location sites of MSM and transgender women in Nepal. Each seed received three coupons for referral and one reward coupon. The referral coupons had unique ID numbers to be able to track recruiters and recruits. Participants recruited by seeds who enrolled in the study were given 3 coupons for further recruitment. Out of the nine seeds, three seeds generated five recruitment waves, whereas six seeds generated four waves. Respondents were given a financial incentive of 300 Nepalese rupees for participation and 200 Nepalese rupees as a reward for each additional participant they recruited. The IBBS survey was initiated in April 2016 and completed in August 2016. Both biological and behavioural data were collected in June 2016.

### Sample size

The following formula was used to calculate sample size in each round of IBBS surveys to detect a 15% change in key indicators [25]:

$$ n=D\frac{{\left[{Z}_{1-\alpha}\sqrt{2 Phat\left(1- Phat\right)}+{Z}_{1-\beta}\sqrt{P_1\left(1-{P}_1\right)+{P}_2\left(1-{P}_2\right)}\right]}^2}{{\left({P}_2-{P}_1\right)}^2} $$

*n* =Sample size required per survey round; D = Design effect of 2; ^*Z*^1-α = The *z* score for the desired confidence level, 1.960 for 95% confidence level for a two-sided test;^*Z*^1-β = The *z* score for the desired power, 0.84 for 80%; P_1_ = the estimated proportion at the time of the first survey (it was .78 for non-use of condoms at their last anal sex with another man and .51 for knowledge of HIV/AIDS etc.); *P*_2_ = The magnitude of change we want to be able to detect, in our case it is 15%; *Phat* = (*P*_1_ + *P*_2_)/2. The sample size per region was determined based on consulting with the BDS and other NGO’s working in the Terai area.

### Measurements

Behavioural data were collected using a structured questionnaire that included questions related to socio-demographic characteristics, sexual risk behaviour, knowledge on HIV and STIs, mobility, experiences of stigma and discrimination, and health service uptake. The questionnaire was pre-tested prior to the survey and a pilot to assess the survey tools was conducted. The questionnaire used can be found in the published IBBS report [23]. The primary outcome variables were HIV and syphilis prevalence. The secondary outcome variables were sexual risk behaviour as assessed by condom use in last sexual encounter and health service uptake which was measured by the question “Have you visited or been to any outreach centre (Drop-In Centre (DIC), Information Centre (IC), Counseling Centre (CC)) in the last 12 months?”. HIV knowledge was assessed by computing a knowledge score which was dichotomized into low and high knowledge. The variables selected for the generation of the score were based on previously published studies from Nepal [26, 27] and consisted of: 1) Can people reduce their risk of HIV by using a condom correctly every time they have sex?; 2) Can a person get HIV from mosquito bites?; 3) Can people protect themselves from HIV by having one uninfected faithful sex partner?; 4) Can people protect themselves from HIV by abstaining from sexual intercourse?; 5) Can a person get HIV by sharing meal with someone who is infected?; 6) Can a person get HIV by using a needle that is used by someone else?; 7) Do you think that a healthy-looking person can be infected with HIV, the virus that causes AIDS?; 8) Can a pregnant woman infected with HIV transmit the virus to her unborn child?; 9) Can women with HIV transmit the virus to her newborn child through breastfeeding?. Discrimination was assessed based on the Experience of Discrimination (EOD) tool [28], which entails a self-reported experience of discrimination in eight different situations, in addition to the reaction to being treated unfairly. The eight situations included school, getting hired, work, getting housing, getting medical care, getting service in store/ restaurant, public settings, and from police or other security personnel and has previously been used in the Nepalese setting [21]. “No response”/“Don’t remember”/“Don’t know” were set to missing.

### Laboratory methods

After completion of the questionnaire, participants were tested for HIV and syphilis according to Nepal’s HIV testing and counselling protocol. The survey participants underwent counselling prior to and post blood sample collection, along with a clinical examination for STIs symptoms and a basic health examination. On-site rapid screening for HIV1/2 was performed using Determine HIV 1/2 (Abbott, Japan). Reactive samples were confirmed using Uni-Gold HIV ½ (Trinity Biotech, Ireland). Samples that were negative on the first test were considered HIV negative, while samples that tested positive on the two first initial tests were considered HIV positive. Any discrepant results from the two first tests were retested with Stat-Pak HIV 1/2 (Chembio Diagnostic system, Inc. USA). Syphilis was tested using Rapid Plasma Reagin (RPR) test (Becton, Dickson, and company, USA). If the first test was positive the sample was retested using Serodia Treponema Pallidum Particle Agglutination (TPPA) test (Fujirebio Inc.). Based on the RPR test a titer value ≥1:8 was categorized as current active syphilis infection, while a titer of < 1:8 was considered as having a history of syphilis. Symptomatic treatment for STIs was given to respondents following the National STI Case Management Guidelines [23].

### Statistical analysis

Data analysis was performed using IBM SPSS Statistics for Windows, Version 24.0 (Armonk, NY: IBM Corp.) and STATA version 14 (StataCorp, College Station, Texas, USA). Data were summarized with descriptive statistics (mean, median, and standard deviation for numerical variables, frequencies and percentages for categorical variables) using both RDS-adjusted and unadjusted values. Values were adjusted for the RDS sampling method using the RDS II estimator, more specifically, weights were established based on the personal network characteristics of each participant. All the RDS-related estimates were adjusted to represent the structure of the study population which is based on information regarding who recruited whom, and the relative size of the respondent’s network using the Volz– Heckathorn estimator (RDS II). The following network size questions were asked to study participants: “How many other MSM and transgender women do you know who also know you well?” (knowing someone is defined as being able to contact them, and having had contact with them in the past 12 months); and “How are you related with the person who gave you the coupon for taking part in the study?” (response category: close friend, friend, sex partner, relative and stranger).

A correct answer to one of the HIV knowledge questions was given the value 1, while a wrong answer was given 0. Scores between 0 and 5 were categorised as low knowledge, while scores of 6–9 were categorised as high knowledge. A discrimination score was generated indicating discrimination in any of the eight situations and dichotomized into never experienced discrimination and experienced discrimination in one or more situations. Bivariate associations between independent categorical variables and sexual risk behaviour and health care service uptake were calculated using Chi-Square or Fisher’s exact test. For independent numerical variables, Mann Whitney U test was used to compare medians in two groups. *P*-values of < 0.25 were set as the criterion for inclusion in the regression model. Both backward and forward stepwise logistic regression models, as well as manual entering of the variables in the model, were used to identify significant predictors of binary outcomes. All methods used gave the same final results. Entry level into the model was set to 0.1, while exit was 0.11. Crude odds ratio (OR) and adjusted odds ratios (AOR) with their 95% confidence intervals (CI) are presented. A *p*-value < 0.05 was considered significant in the final models.

## Results

### General characteristics of the study sample

Sociodemographic characteristics of the study participants stratified by MSM and transgender women are shown in Table 1. Out of the 340 respondents, 173 (61%) identified as transgender women. The mean age (±SD) among MSM was 26.1 (±9.2) years whereas the mean age among transgender women was 31.3 (±10.5). Among the 167 respondents that identified as MSM, 20% were sex workers, whereas in the transgender women group 76% reported being sex workers. Out of the respondents that identified as MSM, 90% reported to have attended school, whereas only 21% of transgender women had received any education. Almost 1/4 of MSM were married, while among transgender women 40% reported being married. Among the MSM and transgender women who were married, the majority reported to be married to a woman (Additional file 1: Table S1). Out of the 340 MSM and transgender women, 120 were residents of the eastern region, 120 were from the western region, and 100 from the far-western region.

**Table 1:** Socio-demographic characteristics among MSM and transgender women in Terai highway districts of Nepal

	MSM, *n* = 167, n (%)		Transgender women, *n* = 173, n (%)		Total, *n* = 340, n (%)	
	Unweighted	Weighted %^a^	Unweighted	Weighted %^a^	Unweighted	Weighted %^a^
Age distribution						
Mean (SD) age	27.4 (9.2)	26.1	31.8 (10.5)	31.3	29.7 (10.0)	28.1
Median age	24		29		27	
Minimum	17		17		17	
Maximum	56		66		66	
Education						
Never attended school	21 (12.6)	10	42 (24.3)	78.8	63 (18.5)	14.3
Ever attended school	146 (87.4)	90	131 (75.7)	21.2	277 (81.5)	85.7
Sexual identity						
Masculine appearing	159 (95.2)	98.3	24 (13.9)	12.6	183 (53.8)	64.6
Feminine appearing	8 (4.8)	1.7	149 (86.1)	87.4	157 (46.2)	35.4
Marital status						
Unmarried	116 (69.5)	77.2	104 (60.1)	60.4	220 (64.7)	29.4
Married	51 (30.5)	22.8	69 (39.9)	39.6	120 (35.3)	70.6
Living situation						
No home/rented	41 (24.6)	23.5	45 (26.0)	26.3	86 (25.3)	24.6
Own home/resident. Hotel	126 (75.4)	76.5	128 (74.0)	73.7	254 (74.7)	75.4
Sex work						
Non sex workers	118 (70.7)	79.7	44 (25.4)	23.8	162 (47.6)	57.7
Sex workers	49 (29.3)	20.3	129 (74.6)	76.2	178 (52.4)	42.3
Income level						
Less than 3000	50 (29.9)	44.5	34 (19.7)	26.1	84 (24.7)	37.3
3000–10,000	73 (43.7)	38.5	74 (42.8)	38.0	147 (43.2)	38.3
Above 10,000	44 (26.3)	17.0	65 (37.6)	35.9	109 (32.1)	24.4
Resident region						
Eastern region	61 (36.5)	35.4	59 (34.1)	42.6	120 (35.3)	38.3
Western region	53 (31.7)	16.3	67 (38.7)	31.6	120 (35.3)	22.2
Far west region	53 (31.7)	48.3	47 (27.2)	26.8	100 (29.4)	39.5

^a^Weighted value based on RDS II Estimator

### HIV and syphilis prevalence among MSM and transgender women

**Fig. 1:**
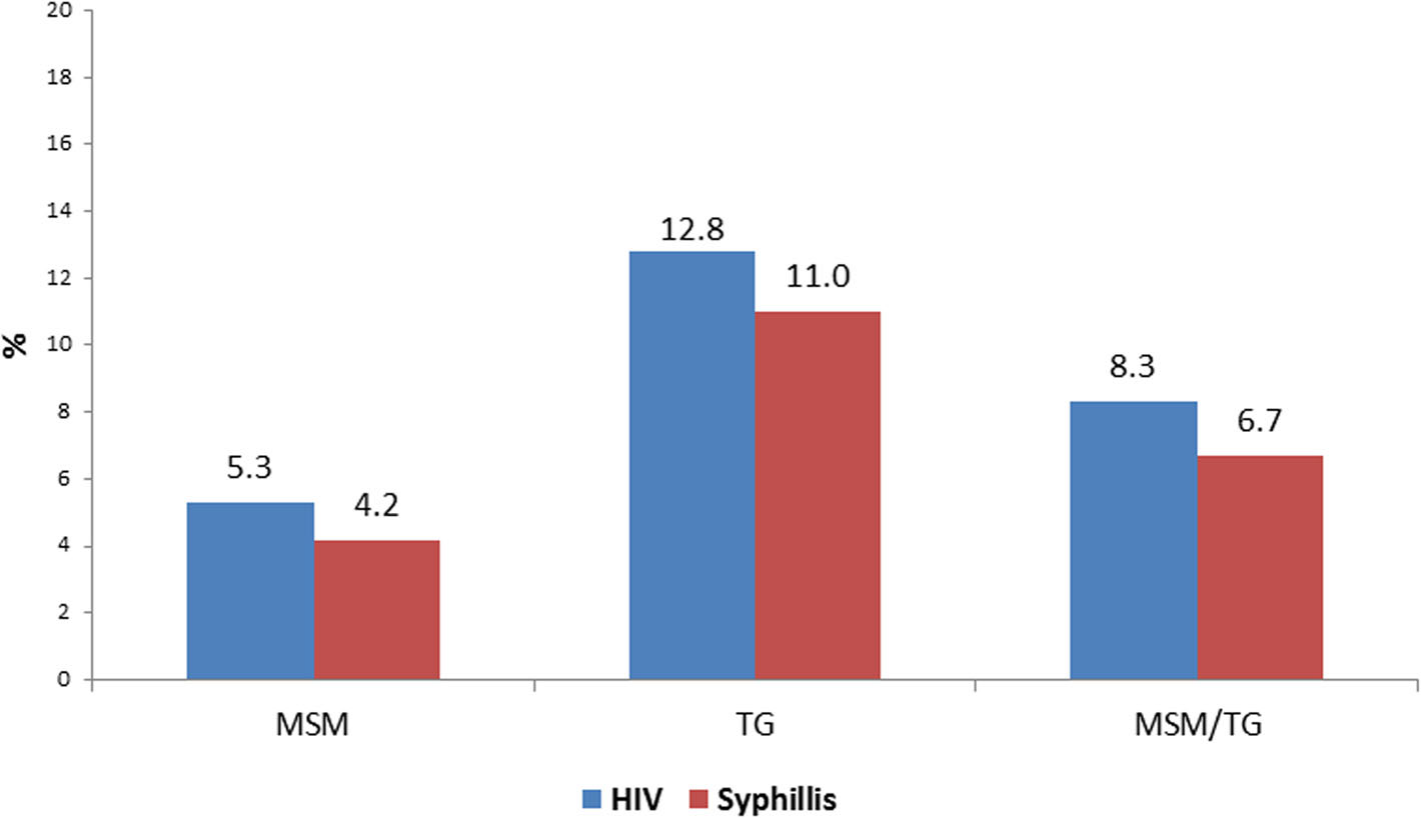
Prevalence of HIV and syphilis among MSM and transgender women in eight Terai highway districts

The prevalence of HIV among MSM was 5%, whereas it was 13% among transgender women. The prevalence of active syphilis was 4% in the MSM group and 11% among transgender women (Fig. 1). The prevalence of HIV and syphilis per region for MSM and transgender women is given in Additional file 2: Table S2. The prevalence of HIV was 2% in the eastern and far-western area, whereas it was slightly higher in the western district (4%). Moreover, the prevalence of syphilis varied across the regions with a prevalence of 4% in the eastern region, 1% in the western region and 2% in the far-western region.

### Sexual risk behaviour, HIV knowledge, socio-structural factors and uptake of HIV services

Among MSM, 61% reported to have used condoms in their last sexual intercourse (Table 2). When comparing condom use across the different regions of the Terai, 91% of MSM and transgender women in the western region reported to have used a condom in the last sexual intercourse, whereas it was 86% in the far-western region, and 77% in the eastern region (Additional file 2: Table S2). When asked who their last sexual partner was 48% MSM and 54% transgender women reported that their last sexual intercourse was with a non-paying male partner. A relatively high proportion of MSM (81%) and transgender women (76%) had a high level of knowledge of HIV risk and transmission. In the MSM group, 25% reported that their family forced them to marry a female and 30% had been away from their home the past month. Among transgender women, 37% reported that their family forced them to marry a female, and 51% had experienced discrimination in one or more situations. In the transgender women group, 55% had interacted with PE/OE/CE/CM and 54% had visited outreach centre in the past year. The uptake of HIV testing and counselling (HTC) services among MSM and transgender women was very low, only 13% of MSM and 22% of transgender women had visited an HTC centre in the last 12 months. Moreover, the uptake of health services varied across the regions. In the eastern region, 20% had visited outreach centre the past year, whereas it was only 14 and 11% in the far-western and western regions, respectively (Additional file 2: Table S2).

**Table 2:** Sexual risk behavior, HIV knowledge, socio-structural factors, and uptake of HIV prevention services among MSM and Transgender women in Terai highway districts

	MSM, *n* = 167, n (%)		Transgender women, *n* = 173, n (%)		Total, *n* = 340, n (%)	
	Unweighted	Weighted %^a^	Unweighted	Weighted %^a^	Unweighted	Weighted %^a^
Sexual risk behavior						
Condom use at last sexual intercourse						
No	69 (41.3)	38.8	76 (43.9)	58.2	145 (42.6)	46.4
Yes	96 (57.5)	61.2	96 (55.5)	41.8	192 (56.5)	53.6
No response	2 (1.2)		1 (0.6)		3 (0.9)	
Last sexual partner						
Non-paying male partner	80 (47.9)	47.9	87 (50.3)	54.1	167 (49.1)	50.3
Non-paying female partner	20 (12.0)	20.3	12 (6.9)	7.7	32 (9.4)	15.3
Male client	46 (27.5)	16.4	61 (35.3)	31.8	107 (31.5)	22.5
Female client	7 (4.2)	8.3	2 (1.2)	1.2	9 (2.6)	5.5
Paid male sex worker	4 (2.4)	1.9	9 (5.2)	3.4	13 (3.8)	2.5
Paid female sex worker	6 (3.6)	4.7	1 (0.6)	1.2	7 (2.1)	3.3
No response	4 (2.4)	0.5	1 (0.6)	0.5	5 (1.5)	0.5
Knowledge level of HIV						
Low knowledge	30 (18.0)	19.3	31 (17.9)	23.7	61 (17.9)	21.0
High knowledge	137 (82.0)	80.7	142 (82.1)	76.3	279 (82.1)	79.0
Socio-structural factors						
Forced by family to marry female						
No	112 (67.1)	75.4	98 (56.6)	63.1	210 (61.8)	70.6
Yes	55 (32.9)	24.6	73 (42.2	36.9	128 (37.6)	29.4
No Response			2 (1.2)		2 (0.6)	
Forced to leave home due to sexual behavior						
No	148 (88.6)	91.0	142 (82.1)	87.5	290 (85.3)	89.4
Yes	19 (11.4)	9.0	31 (17.9)	12.5	50 (14.7)	10.6
Openness to family about sexual behavior						
No	142 (85.0)	93.7	115 (66.5)	22.6	257 (75.6)	87.3
Yes	25 (15.0)	6.3	58 (33.5)	77.4	83 (24.4)	12.7
Beaten due to sexual behavior past year						
No	157 (94.0)	95.7	153 (88.4)	94.9	310 (91.2)	95.4
Yes	10 (6.0)	4.3	20 (11.6)	5.1	30 (8.8)	4.6
Forced to have sex past year						
No	144 (86.2)	93.1	141 (81.5)	82.7	285 (83.8)	89.0
Yes	23 (13.8)	6.9	32 (18.5)	17.3	55 (16.2)	11.0
Cheated/threatened due to sexual behavior in past year						
No	139 (83.2)	93.9	125 (72.3)	78.9	264 (77.6)	88.0
Yes	28 (16.8)	6.1	47 (27.2)	21.1	75 (22.1)	12.0
Don’t Remember/Know			1 (0.6)		1 (0.3)	
Discrimination level						
No discrimination	99 (59.3)	72.3	81 (46.8)	49.0	180 (52.9)	63.9
In one or more situations	68 (40.7)	27.7	92 (53.2)	51.0	160 (47.1)	36.1
Reaction when treated unfairly						
Accept it/keep to self	62 (37.1)	35.5	55 (31.8)	34.8	117 (34.4)	35.2
Do something/keep to self	40 (24)	26.6	60 (34.7)	30.0	100 (29.4)	28.0
Do something/talk to others	65 (38.9)	37.9	58 (33.5)	35.2	123(36.2)	36.8
Away from home last month						
No	120 (71.9)	69.6	113 (65.3)	71.1	233 (68.5)	70.3
Yes	47 (28.1)	30.4	60 (34.7)	28.9	107 (31.5)	29.7
Cross border travel for sexual activities						
No	136 (81.4)	88.9	138 (79.8)	79.7	274 (80.6)	85.3
Yes	30 (18)	11.1	35 (20.2)	20.3	65 (19.1)	14.7
No response	1 (0.6)				1 (0.3)	
Interacted with PE, OE or CM or CE last 12 months						
No	75 (44.9)	40.4	58 (33.5)	45.4	133 (39.1)	45.9
Yes	92 (55.1)	59.6	113 (65.3)	54.6	205 (60.3)	54.1
No response			2 (1.2)		2 (0.6)	
Visited outreach center in the last 12 months						
No	95 (56.9)	75.1	54 (31.2)	45.8	149 (43.8)	63.6
Yes	72 (43.1)	24.9	119 (68.8)	54.2	191 (56.2)	36.4
Visited STI clinic in the last 12 months						
No	146 (87.4)	92.6	143 (82.7)	86.6	289 (85.0)	90.3
Yes	21 (12.6)	7.4	30 (17.3)	13.4	51 (15.0)	9.7
Visited HTC center in the last 12 months						
No	128 (76.6)	87.4	128 (74.0)	78.5	256 (75.3)	83.9
Yes	39 (23.4)	12.6	45 (26.0)	21.5	84 (24.7)	16.1

^a^Weighted value based on RDS II Estimator

### Individual and socio-structural factors associated with sexual risk behaviour in MSM and transgender women

Among MSM, the multiple logistic regression model revealed a negative association between having an income above 10,000 Nepalese rupees and condom use in the most recent sexual encounter (AOR: 0.36, 95% CI:0.14–0.92, *p* = 0.032) (Table 3). Among transgender women, being a resident of the western region was positively associated with condom use in the last sexual intercourse (AOR: 2.74, 95% CI: 1.18–6.39, *p* = 0.020) (Table 4). There was a negative association between being forced by family to marry a female and condom use during last sexual intercourse among MSM (AOR: 0.42, 95% CI: 0.19–0.95, *p* = 0.037) (Table 3). Moreover, respondents that identified as transgender women and reported being away from home the past month were 2.4 more likely to have used condoms in their last sexual intercourse (AOR: 2.43, 95% CI: 1.15–5.10, *p* = 0.019) (Table 4). Transgender women that had a moderate response to being treated unfairly were less likely to have used condom in their last sexual encounter (AOR: 0.39, 95% CI: 0.16–0.92, *p* = 0.032). In multivariable analysis, having visited an outreach centre was positively associated with condom use in the most recent sexual encounter among both MSM (AOR: 5.37, 95% CI: 2.42–11.94, *p* < 0.001) and transgender women (AOR: 2.37, 95% CI: 1.12–5.02, *p* = 0.025) (Tables 3 and 4).

**Table 3:** Logistic regression model of individual and socio-structural factors associated with condom use at last sex among MSM in Terai districts of Nepal, (*n* = 165, missing = 2)

	Crude OR	95% CI	*P*-value	AOR	95% CI	*P*-value
**Individual factors**						
**Age**	0.97	0.94–1.01	0.140			
**Income level**						
Less than 3000	1			1		
3000–10,000	0.52	0.24–1.11	0.091	0.56	0.24–1.28	0.168
Above 10,000	0.43	0.18–1.01	0.052	0.36	0.14–0.92	**0.032**
Total						
**Living situation**						
No home/rented	1			1		
Own home/resident. Hotel	1.90	0.93–3.87	0.078	1.95	0.90–4.25	0.092
**Syphilis**						
Negative	1					
Positive	2.56	0.68–9.67	0.166			
**Socio-structural factors**						
**Forced by family to marry female**						
No	1			1		
Yes	0.68	0.35–1.31	0.251	0.42	0.19–0.95	**0.037**
**Cheated/threatened due to sexual behaviour in past year**						
No	1					
Yes	2.01	0.83–4.87	0.124			
**Away from home last month**						
No	1					
Yes	0.52	0.26–1.04	0.063			
**Interacted with PE, OE or CM or CE last 12 months**						
No	1					
Yes	2.40	1.28–4.53	0.007			
**Visited outreach centre in the last 12 months**						
No	1			1		
Yes	3.35	1.71–6.55	< 0.001	5.37	2.42–11.94	**< 0.001**

**Table 4:** Logistic regression model of individual and socio-structural factors associated with condom use at last sex among transgender women in Terai districts of Nepal, (*n* = 172, missing = 1)

	Crude OR	95% CI	*P*-value	AOR	95% CI	*P*-value
**Individual factors**						
**Income level**						
Less than 3000	1					
3000–10,000	2.51	1.09–5.77	0.031			
Above 10,000	2.36	1.01–5.54	0.048			
Total						
**Resident region**						
Eastern region	1			1		
Western region	3.63	1.72–7.67	0.001	2.74	1.18–6.39	**0.020**
Far west region	1.30	0.60–2.82	0.500	1.42	0.63–3.24	0.402
**Active syphilis**						
Negative	1					
Positive	0.49	0.19–1.26	0.136			
**Syphilis**						
Negative	1					
Positive	0.55	0.22–1.39	0.206			
**Socio-structural factors**						
**Forced by family to marry female**						
No	1			1		
Yes	0.65	0.36–1.21	0.175	0.49	0.24–1.02	0.057
**Reaction when treated unfairly**						
Accept it/keep to self	1			1		
Do something/keep to self	0.31	0.14–0.68	0.004	0.39	0.16–0.92	**0.032**
Do something/talk to others	0.30	0.13–0.66	0.003	0.43	0.18–1.04	0.061
**Away from home last month**						
No	1			1		
Yes	1.72	0.90–3.28	0.103	2.43	1.15–5.10	**0.019**
**Visited outreach centre in the last 12 months**						
No	1			1		
Yes	1.85	0.96–3.56	0.065	2.37	1.12–5.02	**0.025**

### Individual and socio-structural factors associated with visiting outreach Centre among MSM and transgender women

In the previous multivariable regression models for MSM and transgender women, having visited an outreach centre in the past year was significantly associated with sexual risk behaviour (Tables 3 and 4). The determinants of health care service uptake among MSM and transgender women were thus further examined. In the final model having a high level of knowledge of HIV risk and transmission (AOR: 3.33, 95% CI: 1.22–9.11, *p* = 0.019) was positively associated with having visited an outreach centre in the last 12 months among MSM (Additional file 3: Table S3). MSM who reported that they had been forced to marry a female were 3 times more likely to have visited an outreach centre (AOR: 3.08, 95% CI: 1.49–6.38, *p* = 0.002). Moreover, having been cheated or threatened due to sexual behaviour (AOR: 3.87, 95% CI: 1.45–10.36, *p* = 0.007) was associated with increased health care service uptake. Multivariable regression revealed that transgender women with comprehensive knowledge on HIV were more likely to have visited outreach center (AOR: 6.03, 95% CI: 2.48–14.66, *p* < 0.001) (Additional file 4: Table S4). Furthermore, openness to family about sexual behaviour (AOR: 2.40, 95% CI: 1.04–5.57, *p* = 0.041) and having been cheated or threatened due to sexual behaviour (AOR: 2.36, 95% CI: 0.95–5.89, *p* = 0.065) were positively associated with having visited outreach center.

## Discussion

This study assessed the HIV and syphilis prevalence along with factors that contribute to sexual risk behaviour and health care service uptake among MSM and transgender women in Terai highway districts of Nepal. In this study, the prevalence of HIV among MSM was 5%, whereas it was 13% in transgender women. The prevalence of syphilis was also markedly higher among transgender women (11%) compared to MSM (4%). Furthermore, the prevalence of HIV among MSM and transgender women was slightly higher in the western region, compared to the two other regions. In contrast, the prevalence of syphilis was somewhat higher in the eastern region, compared to the western and far-western regions. Overall, transgender women had a higher prevalence of socio-structural factors concerning discrimination and marginalisation compared to MSM. These results are consistent with previous studies which have shown that transgender women bear a substantial part of the HIV burden compared to MSM, and that transgender women face considerable stigma and discrimination [4, 29]. Furthermore, these findings highlight the importance and need for disaggregation of data by gender identity both in terms of research, but also monitoring.

In this study, approximately ¾ of transgender women were involved in sex work. Due to the stigma and discrimination transgender people experience they often have difficulties finding employment and thus resort to sex work as a means of income [30]. Being engaged in sex work can result in additional stigma and increased HIV vulnerability. Future initiatives should address the aspect of stigma and employment among transgender women in Nepal. Overall, the prevalence of HIV and syphilis was higher in this survey than what was observed in the latest IBBS surveys conducted among MSM and transgender women in the Kathmandu valley [7, 31]. This could be due to, at least in part, its location, the Terai highway district area. The open border with India, high population density, as well as sex work and drug trafficking across the border makes this area particularly vulnerable to HIV and STIs spread [12]. Out of respondents that identified as MSM, 61% reported to have used condom in their last sexual intercourse, whereas only 42% of transgender women had used a condom in last sex. Since a relatively high percentage of both MSM and transgender women reported being married, and the last sexual intercourse could thus refer to a female spouse this could potentially cloud the results. However, as the majority of both MSM and transgender women reported that their last sexual partner was male this indicates that condom use in this setting refers to condom use with male partner.

### Individual and socio-structural factors associated with sexual risk behaviour among MSM and transgender women

Respondents that identified as MSM and had an income above 10,000 Nepalese rupees had a lower likelihood of using a condom in the last sexual encounter compared to respondents that earned less. Studies have shown both low and high income to be associated with more unprotected sex and the underlying reason behind the observed association is not clear [32–34]. Controlling for the other covariates in the model, being forced by family to marry a female was associated with lack of condom use during the last sexual encounter among MSM. This increased sexual risk behaviour can, on the one hand, be seen as a product of the stigmatisation and discrimination that MSM and transgender women in Nepal experience and is consistent with the proposed pathway in which how structural violence such as stigma leads to sexual risk behaviour [14]. Studies from China and Cambodia have highlighted the strong societal and familial pressures on LGBT to marry and suggested it can lead to increased sexual risk behaviours [35–37]. Family rejection and pressure to marry can cause psychosocial problems and also result in MSM and transgender people to run away from home which leads to increased sexual risk behaviour, including engaging in survival sex [18, 37]. However, this association could also be explained by that respondents who report to be forced by family to marry are in fact married and thus less likely to use condoms in sexual encounters with their wives. Moreover, among both MSM and transgender women, having visited an outreach centre was positively associated with condom use during the most recent sexual encounter. This indicates that the outreach services are effective in reducing sexual risk behaviour and emphasises health care service uptake as an important deterrent to sexual risk behaviour among MSM and transgender women in Nepal. This is also consistent with an earlier study among MSM and transgender women in Nepal that showed that non-participation in HIV prevention services was associated with decreased condom use in the last three anal sex encounters [19].

Multivariable regression revealed that being away from home the past month was associated with increased condom use among transgender women. As a considerable portion of the respondents that identified as transgender women was involved in sex work, this association could be explained by mobility in connection with sex work or survival sex and a subsequent increase in condom use. Sex work is often connected to mobility and sexual risk behaviour and, transgender women involved in sex work have been found to be less likely to have unprotected sex with clients compared to stable partners [38, 39]. The response to unfair treatment is thought to be a measurement of coping, i.e. how an individual adapts to a problematic situation [28, 40], and is here categorised into active, moderate, and passive. An active response was defined as talking to others and acting by trying to do something about it. A moderate response was talking to someone about it but did not act, or did not talk to anyone but acted. A passive reaction was defined as not talking to anyone and not acting [28, 40]. In the model, having a moderate response to unfair treatment was associated with a decreased odds of condom use compared to a passive response. This result could be interpreted as that a more accepting and less outspoken coping strategy to unfair treatment in which transgender women try to comply with the recommended guidelines and does not dare to speak out is associated with condom use, whereas an active response could signify empowerment and engagement in sexual risk behaviour. However, several different definitions of coping strategies have been proposed and it is not clear how these relate to unprotected sex and discrimination in different populations and contexts [41]. This is also a difficult concept to tap into and requires a well-designed culturally appropriate instrument [41]. For transgender women, being a resident in the western region was associated with increased condom use compared to residing in the eastern and far-west region. This result also coincides with what was observed in the regional analysis of condom use in last sex, in which condom use in last sex among MSM and transgender women who resided in the western region was higher than the other two regions. The underlying reasons behind this association is not clear, there does not seem to be any reports showing regional differences in interventions for MSM/transgender women. The number of HTC sites and services are approximately the same in the different regions that were surveyed [42].

### Individual and socio-structural factors associated with visiting outreach Centre among MSM and transgender women

Given that having visited an outreach centre was significantly associated with sexual risk behaviour among both MSM and transgender women, it was important to examine the factors that influenced uptake of health services. Among MSM, having been forced by family to marry a female was positively associated with visiting an outreach centre. Moreover, both MSM and transgender women who reported to have been cheated or threatened the past year were more likely to have visited an outreach centre. These results could mean that the outreach centres are successful in providing a safe, friendly, and judgment-free setting for the respondents who feel discriminated and marginalised. This is in line with a recent study investigating uptake of HTC services among MSM and transgender women in Nepal which found that MSM and transgender women who experienced forced sex in the past year were more likely to access HTC services [43]. For both MSM and transgender women, there was a positive association between having high level of knowledge on HIV and visiting an outreach centre. This is in line with previous studies which have shown that poor HIV knowledge is associated with decreased HIV testing among MSM and transgender women in Brazil and India [44, 45]. There was a positive association between openness to family about sexual behaviour/identity and having visited an outreach centre. This is consistent with the idea that reduced stigma removes barriers to service uptake and has been observed in a previous study of MSM in Nepal [19].

### Limitations

Given that the survey had a cross-sectional design, no causal inferences can be drawn from the results. Moreover, as the interviews were performed face to face, there is the potential risk of respondents giving socially desirable answers. Since the seeds were recruited through the BDS, this might have limited the representativeness of the data as more hidden groups of MSM and transgender women that are not affiliated with the BDS might potentially have other characteristics. Furthermore, there were also cases of cross recruitment of participants from different areas which led to a smaller number of respondents recruited from the districts Kanchanpur and Kapilbastu than initially planned for [23]. This violation of one of the assumptions of the RDS method may have affected the results of the survey. Moreover, the regional analysis of HIV and STI prevalence, sexual risk behaviour and uptake of health services showed that for certain variables there was some variation among the different regions. However, no specific region stood out as markedly different than the others, indicating that it does not influence the results significantly.

## Conclusions

The high prevalence of HIV and syphilis as well as factors related to discrimination and stigma among transgender women in the Terai highway districts highlights the increased need for surveillance of this population outside the Kathmandu Valley and calls for further action in terms of prevention and treatment for HIV and STI. The results further emphasise the need for disaggregation of data by gender identity, as well as interventions tailored specifically towards the different populations. Given the high HIV/STIs prevalence levels in this area efforts should be made to improve the provision of testing and antiretroviral therapy coverage. Since healthcare service uptake seems to be an important factor in determining sexual risk behaviour among MSM and transgender women in Nepal, outreach services targeting this key population should be scaled up. The BDS, the NGO which is responsible for implementing the majority of interventions targeting MSM and transgender women in Nepal, is run by members of the MSM and transgender people community. The increased condom use associated with outreach centre visits indicates that engagement of the populations can empower them to participate in the provision of services, and thus help raise uptake of services. Furthermore, as the social and structural challenges MSM and transgender women in Nepal experience are encountered by MSM and transgender women worldwide, the results presented here are not only relevant to Nepal, but also generalizable to other settings, especially MSM and transgender women living in rural areas in other low-income countries.

## Supplementary information

**Additional file 1: Table S1.** Marital partner of married MSM and transgender women in Terai area.

**Additional file 2: Table S2.** HIV and STI prevalence, sexual risk behaviour, and uptake of HIV prevention services among MSM and Transgender women per region in the Terai area.

**Additional file 3: Table S3.** Logistic regression model of individual and socio-structural factors associated with visited outreach among MSM.

**Additional file 4: Table S4.** Logistic regression model of individual and socio-structural factors associated with visited outreach centre among transgender women.

## Data Availability

The datasets used and/or analysed during the current study are available from the corresponding author on request.

## Abbreviations

AOR: Adjusted Odds Ratio
BDS: Blue Diamond Society
CC: Community Centre
CE: Community Educators
CI: Confidence Interval
CM: Community Motivators
DIC: Drop-In Centre
EOD: Experience of Discrimination
FSW: Female Sex Worker
HIV: Human Immunodeficiency Virus
HTC: HIV Testing and Counseling
IBBS: Integrated Biological and Behavioural Surveillance
IC: Information Centre
LGBT: Lesbian, Gay, Bisexual and Transgender
MSM: Men who have Sex with Men
MSW: Male Sex Worker
NCASC: National Centre for AIDS and STD Control
OE: Outreach Educator
OR: Odds Ratio
PE: Peer Educator
PWID: People Who Inject Drugs
RDS: Respondent Driven Sampling
STI: Sexually Transmitted Infection
