# Supplementary material for: Prevalence of HIV, syphilis, and assessment of the social and structural determinants of sexual risk behaviour and health service utilisation among MSM and transgender women in Terai highway districts of Nepal: findings based on an integrated biological and behavioural surveillance survey using respondent driven sampling
Source: BMC Infect Dis. 2020 Jun 8;20:402. doi: 10.1186/s12879-020-05122-3 (PMC7282139; doi:10.1186/s12879-020-05122-3)
Supplement: Supplementary file 1 — Additional file 1: Table S1. Marital partner of married MSM and transgender women in Terai area. [file 12879_2020_5122_MOESM1_ESM.docx]

**Supplementary Table 1. Marital partner of married MSM and transgender women in Terai area.**

|  | Married MSM, n=51,  n (%) | | Married transgender women, n=69, n (%) | | Total, n=120, n (%) | |
| --- | --- | --- | --- | --- | --- | --- |
|  | Unweighted | Weighted %* | Unweighted | Weighted %* | Unweighted | Weighted %* |
| Marital partner |  |  |  |  |  |  |
| Man | 8 (15.7) | 2.4 | 11 (15.9) | 4.8 | 19 (15.8) | 3.7 |
| Woman | 41 (80.4) | 94 | 56 (81.2) | 94.8 | 97 (80.8) | 94.4 |
| Transgender woman | 2 (3.9) | 3.6 | 2 (2.9) | 0.4 | 4 (3.3 ) | 1.9 |

*Weighted value based on RDS II Estimator.
