# Supplementary material for: Prevalence of HIV, syphilis, and assessment of the social and structural determinants of sexual risk behaviour and health service utilisation among MSM and transgender women in Terai highway districts of Nepal: findings based on an integrated biological and behavioural surveillance survey using respondent driven sampling
Source: BMC Infect Dis. 2020 Jun 8;20:402. doi: 10.1186/s12879-020-05122-3 (PMC7282139; doi:10.1186/s12879-020-05122-3)
Supplement: Supplementary file 2 — Additional file 2: Table S2. HIV and STI prevalence, sexual risk behaviour, and uptake of HIV prevention services among MSM and Transgender women per region in the Terai area. [file 12879_2020_5122_MOESM2_ESM.docx]

**Supplementary Table 2. HIV and STI prevalence, sexual risk behaviour, and uptake of HIV prevention services among MSM and Transgender women per region in the Terai area.**

|  | **Eastern region** | | **Western region** | | **Far-Western region** | | **Total** | |
| --- | --- | --- | --- | --- | --- | --- | --- | --- |
|  | **MSM and transgender women, n=120, n (%)** | | **MSM and transgender women, n=120, %** | | **MSM and transgender women, n=100, %** | | **MSM and transgender women, n=340, %** | |
|  | Unweighted | Weighted* | Unweighted | Weighted* | Unweighted | Weighted* | Unweighted | Weighted* |
| **HIV** |  |  |  |  |  |  |  |  |
| Negative | 113 (94.2) | 97.6 | 108 (90) | 96.2 | 91 (91) | 97.9 | 312 (91.8) | 91.7 |
| Positive | 7 (5.8) | 2.4 | 12 (10) | 3.8 | 9 (9) | 2.1 | 28 (8.2) | 8.3 |
| **Syphilis** |  |  |  |  |  |  |  |  |
| Negative | 100 (83.3) | 95.8 | 116 (96.7) | 99.2 | 90 (90) | 98 | 306 (90) | 93.1 |
| Positive | 20 (16.7) | 4.2 | 4 (3.3) | 0.8 | 10 (10) | 2 | 34 (10) | 6.9 |
| **Condom use at last sexual intercourse** |  |  |  |  |  |  |  |  |
| No | 64 (53.3) | 23.1 | 36 (30) | 9.3 | 45 (45) | 14.1 | 145 (43) | 46.4 |
| Yes | 56 (46.7) | 76.9 | 84 (70) | 90.7 | 55 (55) | 85.9 | 195 (57) | 53.6 |
| **Interacted with PE. OE or CM or CE last 12 months** |  |  |  |  |  |  |  |  |
| No | 45 (37.5) | 82.8 | 41 (34.2) | 89.3 | 49 (49) | 82 | 135 (39.7) | 54.1 |
| Yes | 75 (62.5) | 17.2 | 79 (65.8) | 10.7 | 51 (51) | 18 | 205 (60.3) | 45.9 |
| **Visited outreach center in the last 12 months** |  |  |  |  |  |  |  |  |
| No | 54 (45) | 80 | 47 (39.2) | 89.3 | 48 (48) | 86.2 | 149 (43.8) | 63.6 |
| Yes | 66 (55) | 20 | 73 (60.8) | 10.7 | 52 (52) | 13.8 | 191 (56.2) | 36.4 |
| **Visited STI clinic in the last 12 months** |  |  |  |  |  |  |  |  |
| No | 88 (73.3) | 93.6 | 107 (89.2) | 98.5 | 94 (94) | 98.1 | 289 (85) | 90.2 |
| Yes | 32 (26.7) | 6.4 | 13 (10.8) | 1.5 | 6 (6) | 1.9 | 51 (15) | 9.8 |
| **Visited HTC center in the last 12 months** |  |  |  |  |  |  |  |  |
| No | 99 (82.5) | 97.3 | 89 (74.2) | 95.1 | 68 (68) | 91.6 | 256 (75.3) | 83.9 |
| Yes | 21 (17.5) | 2.7 | 31 (25.8) | 4.9 | 32 (32) | 8.4 | 84 (24.7) | 16.1 |

*Weighted value based on RDS II Estimator.
