# Supplementary material for: Prevalence of HIV, syphilis, and assessment of the social and structural determinants of sexual risk behaviour and health service utilisation among MSM and transgender women in Terai highway districts of Nepal: findings based on an integrated biological and behavioural surveillance survey using respondent driven sampling
Source: BMC Infect Dis. 2020 Jun 8;20:402. doi: 10.1186/s12879-020-05122-3 (PMC7282139; doi:10.1186/s12879-020-05122-3)
Supplement: Supplementary file 3 — Additional file 3: Table S3. Logistic regression model of individual and socio-structural factors associated with visited outreach among MSM. [file 12879_2020_5122_MOESM3_ESM.docx]

|  | **Crude OR** | **CI 95%** | **p-value** | **AOR** | **CI 95%** | **p-value** |
| --- | --- | --- | --- | --- | --- | --- |
| **Individual factors** |  |  |  |  |  |  |
| **Syphilis** |  |  |  |  |  |  |
| No | 1 |  |  |  |  |  |
| Yes | 2.25 | 0.70 - 7.20 | 0.172 |  |  |  |
| **Knowledge level of HIV** |  |  |  |  |  |  |
| Low knowledge | 1 |  |  | 1 |  |  |
| High knowledge | 3.72 | 1.43 - 9.66 | 0.007 | 3.33 | 1.22 - 9.11 | **0.019** |
| **Socio-structural factors** |  |  |  |  |  |  |
| **Forced marriage** |  |  |  |  |  |  |
| No | 1 |  |  | 1 |  |  |
| Yes | 4.00 | 2.02 - 7.91 | <0.001 | 3.08 | 1.49 - 6.38 | **0.002** |
| **Openness to family about sexual behaviour/identity** | | |  |  |  |  |
| No | 1 |  |  |  |  |  |
| Yes | 3.36 | 1.36 - 8.31 | 0.009 |  |  |  |
| **Beaten because of your sexual behavior** | |  |  |  |  |  |
| No | 1 |  |  |  |  |  |
| Yes | 3.30 | 0.82 – 13.25 | 0.092 |  |  |  |
| **Cheated/threatened due to sexual behaviour in past year** | | |  |  |  |  |
| No | 1 |  |  | 1 |  |  |
| Yes | 5.18 | 2.06 – 13.02 | <0.001 | 3.87 | 1.45 - 10.36 | **0.007** |
| **Discrimination** |  |  |  |  |  |  |
| No discrimination | 1 |  |  |  |  |  |
| In one or more situation | 2.18 | 1.16 – 4.10 | 0.015 |  |  |  |
| **Reaction to treated unfairly because of your sexual orientation** | | | |  |  |  |
| Accept it/keep to self | 1 |  |  |  |  |  |
| Do something/keep to self | 0.71 | 0.31 - 1.64 | 0.426 |  |  |  |
| Do something/talk to others | 1.62 | 0.80 - 3.28 | 0.177 |  |  |  |
| **Crossed border for sexual activities** | |  |  |  |  |  |
| No | 1 |  |  |  |  |  |
| Yes | 1.68 | 0.76 – 3.73 | 0.199 |  |  |  |
| **Forced to have sex** |  |  |  |  |  |  |
| No | 1 |  |  |  |  |  |
| Yes | 2.86 | 1.14 - 7.19 | 0.025 |  |  |  |

**Supplementary Table 3. Logistic regression model of individual and socio-structural factors associated with visited outreach among MSM.**
