# Supplementary material for: Prevalence of HIV, syphilis, and assessment of the social and structural determinants of sexual risk behaviour and health service utilisation among MSM and transgender women in Terai highway districts of Nepal: findings based on an integrated biological and behavioural surveillance survey using respondent driven sampling
Source: BMC Infect Dis. 2020 Jun 8;20:402. doi: 10.1186/s12879-020-05122-3 (PMC7282139; doi:10.1186/s12879-020-05122-3)
Supplement: Supplementary file 4 — Additional file 4: Table S4. Logistic regression model of individual and socio-structural factors associated with visited outreach centre among transgender women. [file 12879_2020_5122_MOESM4_ESM.docx]

|  | **Crude OR** | **CI 95%** | **p-value** | **AOR** | **CI 95%** | **p-value** |
| --- | --- | --- | --- | --- | --- | --- |
| **Individual factors** |  |  |  |  |  |  |
| **Income** |  |  |  |  |  |  |
| Less than 3000 | 1 |  |  | 1 |  |  |
| 3000 - 10000 | 1.02 | 0.44 - 2.35 | 0.968 | 0.89 | 0.36 - 2.23 | 0.806 |
| Above 10000 | 2.48 | 0.99 - 6.22 | 0.054 | 2.27 | 0.84 - 6.17 | 0.108 |
| **Education** |  |  |  |  |  |  |
| Never attended school | 1 |  |  |  |  |  |
| Ever attended school | 1.73 | 0.84 - 3.56 | 0.139 |  |  |  |
| **Knowledge level of HIV** | |  |  |  |  |  |
| Low knowledge | 1 |  |  | 1 |  |  |
| High knowledge | 5.78 | 2.52 - 13.25 | 0.000 | 6.03 | 2.48 - 14.66 | **<0.001** |
| **Socio-structural factors** | |  |  |  |  |  |
| **Forced marriage** | |  |  |  |  |  |
| No | 1 |  |  |  |  |  |
| Yes | 2.35 | 1.17 - 4.72 | 0.017 |  |  |  |
| **Openness to family about sexual behavior/identity** | | | |  |  |  |
| No | 1 |  |  | 1 |  |  |
| Yes | 2.21 | 1.05 - 4.62 | 0.036 | 2.40 | 1.04 - 5.57 | **0.041** |
| **Beaten because of your sexual behavior** | | |  |  |  |  |
| No | 1 |  |  |  |  |  |
| Yes | 4.63 | 1.04 – 20.74 | 0.045 |  |  |  |
| **Cheated/threatened due to sexual behaviour in past year** | | |  |  |  |  |
| No | 1 |  |  | 1 |  |  |
| Yes | 2.74 | 1.18 - 6.38 | 0.019 | 2.36 | 0.95 - 5.89 | 0.065 |
| **Forced to leave home due to sexual identity or behavior** | | | | |  |  |
| No | 1 |  |  |  |  |  |
| Yes | 2.13 | 0.82 - 5.54 | 0.122 |  |  |  |

**Supplementary Table 4. Logistic regression model of individual and socio-structural factors**

**associated with visited outreach centre among transgender women.**
